# Evaluation of Functional Outcomes, Survivorship and Complications of Hypoallergenic Fixed-Bearing Medial and Lateral Unicompartmental Knee Arthroplasty: A Minimum 2-Year Follow-Up

**DOI:** 10.3390/jcm14051748

**Published:** 2025-03-05

**Authors:** Federico D’Amario, Umberto Vitale, Ferdinando De Dona, Luca Ruosi, Alessandro Cofone, Mattia Loppini

**Affiliations:** 1 Orthopedic Unit, Humanitas San Pio X, Via Francesco Nava, 31, 20159 Milano, Italy; 2 Department of Biomedical Sciences, Humanitas University, Via Rita Levi Montalcini, 4, Pieve Emanuele, 20072 Milano, Italy; ferdinando.dedona@humanitas.it (F.D.D.); luca.ruosi@humanitas.it (L.R.);; 3 IRCCS Humanitas Research Hospital, Via Manzoni, 56, Rozzano, 20089 Milano, Italy; 4 Department of Orthopaedic Surgery, S. Andrea Hospital, “Sapienza” University of Rome, Via di Grottarossa, 1035/1039, 00189 Roma, Italy

**Keywords:** unicompartmental knee arthroplasty, oxinium, oxidized zirconium, return to sport, patient-reported outcome measures, satisfaction, lateral, survivorship, fixed-bearing, UKA

## Abstract

**Background:** Unicompartmental knee arthroplasty (UKA) is a viable treatment option for patients with isolated knee osteoarthritis. This study evaluated the clinical outcomes of the JII UK (Smith & Nephew, Memphis, TN, USA) hypoallergenic, fixed-bearing UKA implant in a medium cohort of patients undergoing both medial and lateral procedures with a short-term follow-up. **Methods**: A retrospective review was conducted on 257 consecutive patients who underwent primary UKA using the JII UK implant between December 2020 and December 2022. Clinical outcomes were assessed using the Knee Society Score (KSS), Knee Society Function Score (KSFS), Oxford Knee Score (OKS), UCLA Activity Score, Forgotten Joint Score (FJS-12), and satisfaction. Survivorship analysis was performed, and complications were recorded. **Results**: At the 2-year follow-up, the implant survival rate was 99.61%. Statistical significant improvements were observed in all clinical scores, with high patient satisfaction. The mean UCLA Activity Score increased from 4.53 preoperatively to 7.3 at 24 months. **Conclusions**: This study demonstrates promising short-term clinical outcomes for the hypoallergenic fixed-bearing medial and lateral UKA implant, with high patient satisfaction and a low complication rate. Further studies with longer follow-up periods are warranted to confirm these findings.

## 1. Introduction

Osteoarthritis (OA) of the knee is a degenerative joint disease that occurs when the cartilage in the knee joint gradually wears down. This can lead to pain, swelling, stiffness, and decreased mobility. It has been shown by recent studies that the prevalence of knee osteoarthritis worldwide is approximately 15% [1,2].

Its frequency increases with age, female sex, and other described characteristics. It represents a major cause of disability and a source of societal costs in older adults [3].

Although OA is recognized as a tricompartmental disease of the entire knee [4], it is estimated that as much as 50% of the population experience unicompartmental disease. The medial tibio-femoral compartment is the most affected, with an incidence of 27%, followed by the patello-femoral compartment at 18%, and lastly the lateral tibio-femoral compartment at 5% [5].

The vast majority of unicompartmental OA of the knee is located within the medial compartment, but about 10% is represented by osteoarthritis affecting the lateral compartment [6].

There is ongoing debate about the most appropriate treatment for patients with end-stage unicompartmental osteoarthritis, but unicompartmental knee arthroplasty (UKA) has proven to be a viable option for young and active individuals and also for older patients [7,8].

The Italian Arthroplasty Registry records a 22.2% (50,857) prevalence of primary unicompartmental knee arthroplasty (UKA) among all knee surgeries performed from 2007 to 2022. These findings demonstrate a rising trend in the utilization of unicompartmental knee replacements, which had a prevalence of approximately 15% in the preceding years [9].

While UKA has gained popularity thanks to the development of new minimally invasive techniques, its rapid recovery after surgery, and bone stock preservation [10], careful patient selection is paramount to achieving successful outcomes [11].

Through rigorous patient selection and continuous refinement of surgical techniques, UKA has demonstrated excellent long-term clinical outcomes, with high survival rates and significant improvements in patients’ quality of life, with a mean 10-year survival rate of approximately 94% for medial UKAs [12,13,14].

Even for the lateral compartment, which is traditionally considered more challenging, excellent long-term results have been achieved thanks to implant manufacturing advancements and careful patient selection, with a mean 10-year survival rate of approximately 93% for lateral UKAs [15,16,17].

The literature consistently reports higher failure rates for UKA compared to Total Knee Arthroplasty (TKA) [18,19]. Failure etiologies vary between medial and lateral UKAs, with aseptic loosening, progressive osteoarthritis, polyethylene wear, and mobile dislocation being frequently observed [20,21].

Another cause of revision is metal hypersensitivity, which is extensively described in the literature but still remains poorly regulated by guidelines [22,23,24].

A study investigated whether patients experiencing pain after TKA and with metal sensitivity had better outcomes following a revision with a hypoallergenic implant. After sensitivity testing, patients underwent revision TKA with either a hypoallergenic or a standard component. Among those who tested positive for metal sensitivity (78.3%), the majority were found to be sensitive to nickel. Both reactive and non-reactive patients showed significant improvements in their range of motion after the revision surgery. The reactive group experienced a notable 37.8% reduction in pain six weeks post-revision, while the non-reactive group showed a moderate, yet non-significant, improvement in pain reduction [25].

Although the immunological mechanisms involved are not yet fully understood, manufacturers are making significant investments in and actively promoting hypoallergenic components.

A new fixed-bearing implant (Journey II UK Unicondylar Knee System or JUK, Smith & Nephew, Memphis, TN, USA) was launched in 2019. This implant achieves compatibility with a wide range of patients through the presence of components for both the medial and lateral compartments, a feature not always available in other implants, and an increased range of sizes, particularly smaller ones.

The introduction of a simpler instrument set allows for the enhancement and acceleration of the timing of the surgical technique due to the presence of a tibial guide that facilitates re-cutting and the ability to adjust the mediolateral position of the femoral component to allow for greater congruency with the tibial component.

The JII UK implant’s femoral component (Smith & Nephew, Memphis, TN, USA) is composed of Oxinium™ and articulates against a cross-linked polyethylene surface via a round-on-flat bearing design, making it compatible with patients who have metal hypersensitivity.

Oxidized Zirconium (OxZr, Oxinium™, Smith & Nephew, Memphis, TN, USA) is an advanced bearing material, composed of a zirconium alloy core that has undergone a surface transformation to form a thick ceramized surface. This process results in a material with enhanced properties, including minimized wear, increased strength and stability, and resistance to fretting and corrosion [26,27,28].

The previously cited combined characteristics have the potential to offer increased implant survivorship and improved clinical outcomes compared to currently available implants [29,30,31] owing to their greater patient-specific customization.

Following the introduction of a new implant, it is crucial to study its short-term performance to understand the implant’s survivorship and impact on clinical patient outcomes and to identify any potential weaknesses.

The objective of this study was to assess the functional outcomes, evaluate the survivorship, and identify the reasons for the failure of a hypoallergenic, fixed-bearing medial and lateral UKA implant during a short-term follow-up. In particular, this implant can be used in all patients, including those who suffer from metal hypersensitivity. We have chosen to study this implant because it combines the characteristics of universality with a new and functional prosthetic design. All surgeries were conducted by a single surgeon at one institution between 2020 and 2022. This series is a follow-up to a cohort previously described by our group [32].

## 2. Materials and Methods

### 2.1. Patient Selection

Patients’ data for this single-center retrospective study were extracted from the database of Humanitas San Pio X Hospital systems (Milan, Italy). Upon collection, the data were deposited in restricted-access online storage.

Data from UKA procedures, using medial or lateral JII UK implants (Smith & Nephew, Memphis, TN, USA) performed from December 2020 to December 2022, were analyzed.

The data collected from the institutional database included demographic information, the body mass index (BMI), American Society of Anesthesiologists (ASA) class, duration of the surgical procedure, duration of hospitalization, any surgical revisions, and any intra-operative, peri-operative, and post-operative complications.

This study adheres to the ethical principles outlined in the 2013 revision of the Declaration of Helsinki. Prior to data collection, informed consent was obtained from all subjects involved in the study.

### 2.2. Surgical Indication and Inclusion and Exclusion Criteria

From a clinical standpoint, surgical intervention was proposed to patients who met the Kozinn and Scott criteria [33], including a coronal plane deformity of less than 10 degrees of varus and 5 degrees of valgus and a sagittal plane flexion deformity no greater than 15 degrees. Additionally, patients were required to have an intact anterior cruciate ligament, an unaffected contralateral compartment, and patellofemoral changes not exceeding grade II or III on the Ahlbäck classification [34].

Trochlear cartilage damage up to grade IV was permissible, provided it was centrally located.

Patients with neurological diseases, ataxia, symptomatic osteoarthritis in two or more compartments, or those who did not meet the aforementioned deformity criteria were excluded to prevent implant failure due to the progression of contralateral osteoarthritis or potential falls resulting from neurological issues leading to periprosthetic fractures.

Furthermore, surgical intervention was only proposed to patients who radiographically presented with isolated medial or lateral compartment osteoarthritis with a cartilage wear loss of Kellgren–Lawrence grade of 3 or higher [35], or those with spontaneous femoral osteonecrosis visible on X-ray as cartilage damage of Kellgren–Lawrence grade 3 or higher, or on MRI.

The characteristics of patients eligible for this type of surgery, as previously outlined, have been discussed based on the latest evidence from the literature, both for medial and lateral implants [36,37].

Prior to surgical intervention, patients underwent a 3-month course of conservative treatment including physiotherapy, hyaluronic acid or corticosteroid injections, and rest from physical activity. Surgical intervention was proposed only if pain and disability persisted after this conservative treatment period.

The study included patients who underwent a primary medial or lateral UKA using the JII UK (Smith & Nephew, Memphis, TN, USA) implant and had a minimum 2-year follow-up.

Patients were excluded if they had undergone bilateral UKA, concomitant surgical procedures on the same knee (e.g., ACL reconstruction or osteotomy), had a concurrent inflammatory rheumatic disease, had not a minimum 2-year follow-up, or refused the informed consent.

### 2.3. Surgical Procedure

All surgical procedures were performed by a single, high-volume senior surgeon (F.D.A.).

All patients received a prophylactic antibiotic of 2 g of Cefazolin intravenously 30 min prior to the procedure. If the patient was allergic or had a hypersensitivity to penicillins, 600 mg of Clarithromycin was administered intravenously 30 min prior to incision. All surgical procedures were performed under spinal anesthesia, with the addition of an adductor canal block when deemed necessary. No tourniquet was used during any procedure.

**Figure 1:**
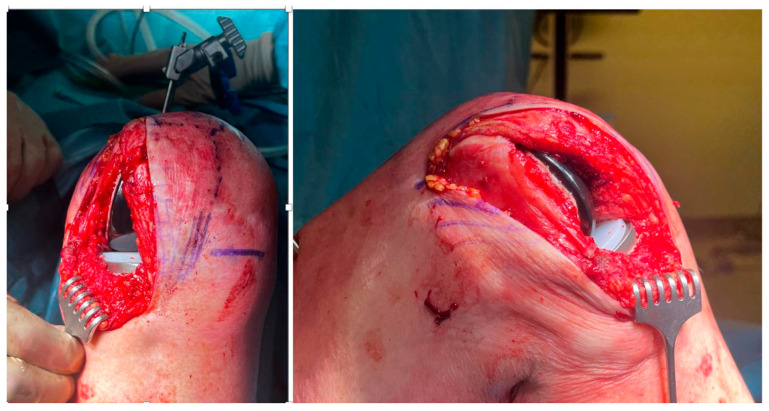
Placement of the lateral tibial and femoral components on both the coronal (on the left) and sagittal (on the right) planes.

While the technique for medial UKAs was extensively described in a previous article [32], there are some differences in the lateral UKA technique. The skin incision in the latter case is in the lateral region of the knee, utilizing a parapatellar approach. The tibial cut was not made along the tibial epiphyseal axis but rather aimed for a cut perpendicular to the mechanical axis or slightly valgus, with a neutral slope (Figure 1). The surgical steps followed the manufacturer’s recommendations. After performing the tibial cut, a trial 8 mm spacer block was used to prevent flexion deformity, and the distal femoral cut was made using a specific template. If the 8 mm trial spacer block does not fully fit within the joint, the instrumentation includes the use of a tibial re-cutting guide. The proximal femoral cut was then completed by aligning the component with the tibial component. Within this surgical instrument set, femoral cutting guides are categorized into groups ranging from 1 to 3, 4 to 7, and 8 to 10. Following the identification of the appropriate size based on the cartilaginous line, the femoral cutting component can be precisely adjusted medially or laterally by approximately 1 to 3 mm to accommodate individual anatomical variations. Once the trial components were positioned and the final sizes determined, the articular surface was roughened with multiple saw cuts to improve cement penetration [32]. Before cementation, the surface was washed with a pulsed lavage to remove debris, and the entire surgical team changed gloves. The prepared cement was applied to both the bone surface and the components. After implantation, excess cement was removed, paying particular attention to the posterior region, the patellofemoral joint, and the contralateral compartment. A trial polyethylene component was used to re-assess stability and limb alignment. If these characteristics were deemed satisfactory by the primary surgeon, the final polyethylene component was implanted. Following hemostasis, the wound was thoroughly irrigated and closed in layers. The deep fascia was sutured with a waterproof material, and the skin was closed primarily with staples or an intradermal suture. When clinically indicated, a 3 g intra-articular injection of tranexamic acid was administered following the closure of the articular capsule. Following surgery, postoperative radiographs were obtained to verify component placement.

### 2.4. Postoperative Care

Adhering to a fast-track protocol, patients were mobilized on the same day and commenced physiotherapy under the guidance of a physiotherapist with partial progressive weight-bearing. As thromboprophylaxis, all patients receive 4000 IU of low-molecular-weight heparin starting 12 h post-surgery and continuing for 5 weeks. Post-operative follow-up includes weight-bearing anteroposterior and lateral radiographs at 1, 3, 6, 12, and 24 months.

A radiographic assessment of the knee joint was conducted utilizing the Knee Society Roentgenographic Evaluation system [38]. This system enabled the evaluation of the femoro-tibial angle and the identification of any radiolucent areas within the femur and tibia on both anteroposterior and lateral radiographic projections.

The decision to revise the surgical intervention was informed by a comprehensive assessment encompassing patient symptomatology, documented medical history, diagnostic imaging results, and intraoperative observations.

### 2.5. Clinical Outcomes

Patient-reported outcome measures (PROMS) were analyzed at baseline, 12, and 24 months. These included the Knee Society Knee Score (KSKS), the Knee Society Function Score (KSFS) [39], the Oxford Knee Score (OKS) [40], and the University of California, Los Angeles Activity Score (UCLA) [41].

Physical Component Summary (PCS) and the Mental Component Summary (MCS) of the Short Form 36 Health Survey (SF-36) [42] and the Forgotten Joint Score-12 (FJS-12) [43] were analyzed too. This last score was collected at 12- and 24-month follow-ups. Patient satisfaction (rated from 1, not satisfied, to 10, completely satisfied) was collected at the last follow-up visit.

### 2.6. Statistical Analysis

All analyses were carried out using R (version 4.4.2, The R Foundation for Statistical Computing, Vienna, Austria). The Shapiro–Wilk test was employed to assess whether the data exhibited a non-parametric distribution. Calculated mean values were provided for all continuous data, while percentage frequencies were used for qualitative variables. Baseline and postoperative clinical scores were compared using the non-parametric Friedman test, a method for repeated measures analysis. This test was applied to evaluate differences in the KSKS, KSFS, OKS, UCLA, PCS, and MCS scores between baseline and the next two time points (12 and 24 months). The test was also applied to evaluate the FJS-12 at 12 and 24 months. A *p*-value < 0.05 was considered statistically significant.

## 3. Results

### 3.1. Demographics

The demographic data are reported in Table 1.

### 3.2. Clinical Outcomes

The clinical and functional outcomes of the patients and the result of the statistical analyses are reported in Table 2.

### 3.3. Complications and Revisions

Analysis of 257 cases revealed a 99.61% implant survival rate at 24 months using re-surgery for any reason as an endpoint. There was a single adverse event related to aseptic loosening of the tibial component. The case of tibial aseptic mobilization concerns a 75-year-old female patient with a BMI of 19. The surgical technique employed was identical to that used in other medial implants. The patient underwent a conversion to TKA 12 months after the UKA.

No additional intraoperative or rehabilitation-related complications were encountered.

## 4. Discussion

The new hypoallergenic fixed-bearing medial and lateral UKA implant provided excellent clinical results with a low rate of complications. Statistical analysis demonstrated highly significant improvements in all clinical outcome scores (*p* < 0.05) between preoperative and postoperative assessments.

The KSKS section, which constitutes the first part of the KSS, highlights how symptoms related to pain and the range of motion in the knee have significantly improved in these patients. On the other hand, the KSFS section reflects an increase in patient functionality, allowing them to walk longer distances and reducing or eliminating the need for assistive devices [39].

The OKS represents a complementary score to the KSS, in which, in addition to pain symptoms and functionality, improvements in daily living activities can also be assessed [40].

The fact that all these scores demonstrated statistically significant improvements suggests that the patients’ quality of life increased compared to their clinical status prior to the surgical intervention.

By preserving a larger portion of the articular surface, UKA allows for more natural and fluid knee motion [36,44,45]. This translates into an improved body image and greater patient satisfaction, as demonstrated by the result of the patients’ satisfaction with a mean of 9.17 ± 1.16 at the last follow-up.

We compared the FJS-12 at 12 months and at the last follow-up of 24 months, demonstrating a statistically significant improvement from 81.64 ± 16.77 to 85.47 ± 15.38 (*p* < 0.05), showing the patients’ successful adaptation to life with the artificial joints. The data obtained in our study are superior to those found in the literature [46,47].

The preservation of bone stock and the reproduction of native-like knee biomechanics results in higher UKAs FJS-12 scores when compared to TKA [48].

The only case of failure we reported was due to aseptic mobilization of the tibial component in a medial unicompartmental prosthesis. The patient was a 75-year-old woman with poor bone quality, in whom early mobilization occurred. Despite the adoption of proper surgical techniques, including good alignment of the components, correct joint line height, and appropriate cementing technique, the patient’s bone quality affected the implant’s survival. Monitoring intraoperative bone quality is a critical factor that may determine the success of the surgical procedure. The patient underwent conversion to TKA approximately one year after the surgical intervention.

The data obtained thus far suggest that our results are at least on par with, and potentially better than, those previously published [49,50,51,52].

Following a comprehensive literature review, only one additional study was identified that evaluated the outcomes of both medial and lateral JUKII [53]. Similar to our study, only primary medial and lateral UKAs performed by high-volume surgeons were retrospectively analyzed. The total cohort comprised 944 UKAs, with 370 reaching at least 1.75 years of follow-up, comparable to our cohort. Using re-operation for any reason as an endpoint, 2-year survival was 98.2%. Of the nine re-operations, however, only three required conversion to TKA. Clinical outcomes demonstrated progressive improvements; however, only 81 patients completed all KOOS assessment stages. While the KOOS showed a positive effect in this subgroup, no statistically significant difference was found compared to patients who did not complete all follow-up stages [53].

It is well-established that engaging in any level of physical activity, even at a low intensity, is a protective factor against many diseases, particularly cardiovascular conditions. Moreover, maintaining adequate muscle tone helps to reduce the incidence of musculoskeletal injuries. In patients with knee osteoarthritis, the role of physical activity is even more pronounced. Given the increasing number of patients undergoing UKA, including younger individuals, the proportion of patients who return to sport (RTS) is a crucial indicator of the procedure’s success, as it also leads to improved PROMs.

A meta-analysis conducted in 2022 revealed that, with a mean follow-up of 48 months, over 90% of patients returned to sports, a finding consistent with our study [54]. RTS was linked to improved PROMs, despite being undertaken at reduced intensities compared to pre-surgery levels [54].

The mean preoperative UCLA score of our patients improved from 4.53 ± 1.6 to 7.3 ± 1.65 after 24 months of follow-up. Similarly, a 2021 study by Panzram et al. demonstrated a statistically significant increase in UCLA scores from a pre-operative mean of 2.9 ± 1.7 to a post-operative mean of 6.3 ± 1.4 at a minimum 2-year follow-up [55]. Furthermore, it is noteworthy that the proportion of high-impact sports activities post-surgery is not substantial, and elevated BMI levels negatively impact these characteristics [55].

A cohort of 85 patients with a minimum follow-up of 2 years found no significant difference in RTS (amateur level) between medial and lateral UKA, aligning with our findings [56]. Consequently, both procedures can be considered for younger patients with high functional demands.

Our cohort consisted exclusively of patients receiving hypoallergenic implants. These findings align with the existing literature, indicating that the selection of hypoallergenic implants does not compromise return to sports (RTS) compared to traditional chromium–cobalt implants or those made of other materials, even in patients with metal hypersensitivity [57,58].

All hypoallergenic implants used in this study had an Oxinium™ component. The study of D’Ambrosi et al. in 2022 did not demonstrate any inferiority of this component compared to other materials used in other UKAs [59].

The use of hypoallergenic materials, which can be implanted in all patients, allows the surgeon to enhance their experience and familiarity with a single implant, thereby increasing the likelihood of proper implant placement and, consequently, achieving better clinical outcomes and improved survival.

Despite the positive outcomes observed with Oxinium™ in our cohort and other studies previously cited, it is important to recognize that the scientific literature also describes adverse clinical cases characterized by complications such as metallosis and polyethylene dislocations both in UKAs and TKAs [60,61,62,63].

This study has several limitations. Firstly, conducted at a high-volume tertiary referral center, its findings may not be generalizable to institutions with lower UKA volumes. Second is the short follow-up duration. While the 2-year follow-up period facilitates the evaluation of short-term effects, it remains insufficient for the comprehensive assessment of long-term effects. Longer duration studies will be necessary to assess long-term outcomes and survival. Furthermore, the absence of a control group (e.g., a conventional metal prosthesis group) may have influenced the interpretation of the results. Randomized controlled trials (RCTs) should be conducted to more accurately assess the differences between these two prosthetic implants.

## 5. Conclusions

Our study of the new Oxinium™ metal-backed fixed-bearing medial and lateral UKA implant demonstrated favorable clinical outcomes along with excellent 2-year survival rates, successful return to sports activity, and high patient satisfaction. Oxinium™ has proven to be a reliable material for all patients, which can be used regardless of the allergic profile without any adverse effects. Nevertheless, good results in terms of clinical outcomes and survival can only be achieved through proper indications and excellent surgical techniques. Furthermore, randomized controlled trials with extended follow-up periods are necessary to validate these findings with a higher level of evidence.

## Figures and Tables

**Table 1:** Key data of the 257 UKA patients.

Variable	Patients
Age, mean (SD) (range)	67.46 (10.33) (32–96)
Gender	
Male (%)	107 (41.80%)
Female (%)	150 (58.37%)
BMI (kg/m^2^), mean (SD) (range)	26.58 (4.61) (16.33–39.26)
ASA class (%)	
ASA 1	59 (22.96%)
ASA 2	159 (61.87%)
ASA 3	39 (15.18%)
Operative time (min), mean (SD) (range)	39.47 (8.56) (20–73)
Side	
Right (%)	124 (48.25%)
Left (%)	133 (51.75%)
Hospital stay (days), mean (SD) (range)	3.95 (1.73) (2–20)

UKA: unicompartmental knee arthroplasty; SD: standard deviation; BMI: body mass index; ASA: American Society of Anesthesiology.

**Table 2:** Preoperative and postoperative clinical and functional data and outcome satisfaction.

	Variable	Patients	*p*-Value
KSKS	Preoperative, mean (SD) (range)	44.53 (14.76) (20–70)	<0.001
	12 months, mean (SD) (range)	86.17 (11.92) (56–99)	
	24 months, mean (SD) (range)	89.31 (10.48) (56–100)	
KSFS	Preoperative, mean (SD) (range)	53.12 (15.6) (15–80)	<0.001
	12 months, mean (SD) (range)	87.78 (10.76) (45–100)	
	24 months, mean (SD) (range)	90.44 (11.08) (45–100)	
PCS	Preoperative, mean (SD) (range)	31.51 (10.65) (6–48)	<0.001
	12 months, mean (SD) (range)	42.54 (10.46) (11–67)	
	24 months, mean (SD) (range)	51.86 (8.78) (26–76)	
MCS	Preoperative, mean (SD) (range)	47.04 (10.44) (15–74)	<0.001
	12 months, mean (SD) (range)	52.35 (10.43) (24–72)	
	24 months, mean (SD) (range)	56.41 (10.11) (19–78)	
OKS	Preoperative, mean (SD) (range)	35.69 (9.13) (6–48)	<0.001
	12 months, mean (SD) (range)	22.43 (7.96) (3–40)	
	24 months, mean (SD) (range)	17.94 (8.19) (0–45)	
FJS-12	12 months, mean (SD) (range)	81.64 (16.77) (15–100)	0.0196
	24 months, mean (SD) (range)	85.47 (15.38) (15–100)	
UCLA	Preoperative, mean (SD) (range)	4.53 (1.6) (1–9)	<0.001
	12 months, mean (SD) (range)	6.3 (1.45) (3–9)	
	24 months, mean (SD) (range)	7.3 (1.65) (3–10)	
Satisfaction	24 months, mean (SD) (range)	9.17 (1.16) (2–10)	

KSKS: Knee Society Knee Score; SD: standard deviation; KSFS: Knee Society Function Score; OKS: Oxford. Knee Society; UCLA: University of California, Los Angeles Activity Score; PCS: Physical Component Summary; MCS: Mental Component Summary; FJS-12: Forgotten Joint Score.

## Data Availability

The datasets generated during the current study are available from the corresponding author upon reasonable request.
